# Assessing early therapeutic response in extrapulmonary tuberculosis using [18F]FDG-PET/CT imaging

**DOI:** 10.3389/fmed.2026.1908921

**Published:** 2026-10-02

**Authors:** Dev Kartan, Ziba Ahangarani Farahani, Søren Hess, Rikke Søndergaard, Oke Gerke, Isik Somuncu Johansen

**Affiliations:** 1 Department of Infectious Diseases, Odense University Hospital, Odense, Denmark; 2 Lewis Katz School of Medicine, Philadelphia, PA, United States; 3 Department of Nuclear Medicine, Odense University Hospital, Odense, Denmark; 4 Research Unit of Nuclear Medicine, Department of Clinical Research, University of Southern Denmark, Odense, Denmark; 5 Research Unit of Infectious Diseases, Department of Clinical Research, University of Southern Denmark, Odense, Denmark

**Keywords:** [18F]FDG, c-reactive protein, extrapulmonary tuberculosis, imaging biomarkers, metabolic response, PET/CT, treatment monitoring

## Abstract

**Introduction:**

Monitoring treatment response in extrapulmonary tuberculosis (EPTB) remains challenging due to limited microbiological accessibility and the lack of validated biomarkers. [18F]FDG positron emission tomography/computed tomography [(18F)FDG-PET/CT] enables assessment of metabolic disease activity and has been explored in infectious diseases, but evidence supporting its use in EPTB is limited.

**Methods:**

We conducted a prospective observational study including adults diagnosed with EPTB at a tertiary referral center in Denmark between 2016 and 2018. Participants underwent baseline and follow-up [18F]FDG-PET/CT imaging after completion of the intensive treatment phase. Clinical data and laboratory parameters, including C-reactive protein (CRP), hemoglobin, leukocytes, albumin, and alanine aminotransferase were collected at baseline and during follow-up. Changes in standardized uptake values (SUVmax) and laboratory markers were analyzed descriptively.

**Results:**

Seventeen patients were included. Metabolically active disease was detected in all participants at baseline. Most patients (11 out of 14) demonstrated a reduction in lesion number and SUVmax at follow-up, indicating a metabolic response to therapy. However, persistent [18F]FDG uptake was observed in a subset of patients (3 out of 14). Median CRP levels declined from 12 mg/L (range: 0.3–190) at baseline to 3.6 mg/L (range: 0.9–20) at week 8 and remained low thereafter, broadly paralleling PET/CT findings. In several cases, residual metabolic activity did not result in treatment prolongation.

**Discussion:**

Serial [18F]FDG-PET/CT demonstrated changes in metabolic activity during treatment with reductions in lesion number and SUVmax in most patients. However, SUVmax reflects focal metabolic activity rather than total disease burden, and persistent uptake after the intensive treatment phase was observed in some patients. Larger prospective studies incorporating standardized response criteria and volumetric PET measures are needed before [18F]FDG-PET/CT can be implemented as a routine biomarker for treatment response in EPTB.

## Introduction

Tuberculosis (TB) remains a major global health concern (1). In 2024, an estimated 10.7 million people developed TB and 1.23 million deaths were attributed to the disease (2). In the European region, extrapulmonary TB (EPTB) accounts for about 20% of TB cases and may reach 30% in certain countries (3). Despite improved diagnostic strategies and access to therapy, EPTB remains challenging due to non-specific clinical presentation, delays in diagnosis, and difficulties in monitoring treatment response (4, 5). Common EPTB sites include lymph nodes, central nervous system (CNS), abdomen, skin, bones, and the gastrointestinal and genitourinary tract (6, 7).

Treatment monitoring is particularly problematic in EPTB because the disease is frequently paucibacillary and often inaccessible for repeated sampling. Conventional diagnostic tools such as smear microscopy and culture have limited sensitivity and microbiological confirmation may be delayed or absent (8). Furthermore, there is currently no validated biomarker that reliably reflects disease activity and treatment response in EPTB. Although inflammatory biomarkers, such as Monokine Induced by Gamma interferon (MIG), Interferon Gamma–Induced Protein 10 (IP-10) and Macrophage Migration Inhibitory Factor (MIF), have shown promise in selected studies, they require further validation before clinical implementation (9). In practice, clinicians often rely on symptom resolution, radiological changes, weight gain and nonspecific inflammatory markers such as C-reactive protein (CRP). However, these measures are imperfect, symptoms may persist despite microbiological cure, structural imaging often lag behind clinical improvement, and inflammatory markers lack specificity for ongoing TB activity (10).

[18F]FDG positron emission tomography/computed tomography [(18F)FDG-PET/CT] is a noninvasive hybrid imaging modality increasingly used in inflammatory and infectious diseases. [18F]FDG uptake reflects increased glucose metabolism in activated immune cells and can provide whole-body mapping of disease extent and metabolic activity (11).

[18F]FDG-PET/CT imaging has demonstrated utility in identifying occult sites of infection, staging multifocal disease, and supporting treatment monitoring in TB, particularly in complex EPTB presentation (12–14). Qualitative visual assessment remains the cornerstone of PET/CT imaging, but advances in PET technology also enable robust quantitative analysis of tracer uptake in tissues. These quantitative metrics may complement or, in some cases, supersede visual interpretation by providing a more objective and precise evaluation of disease burden and treatment response, particularly in systemic conditions. Serial [18F]FDG-PET/CT may allow quantification of changes in these parameters as surrogate markers for therapeutic response also in inflammation (15).

The aim of this study was to investigate the utility of serial [18F]FDG-PET/CT imaging combined with routine laboratory markers for assessing early treatment response in patients with EPTB in Denmark.

## Methods

### Study design and participants

This study was conducted at the Department of Infectious Diseases, Odense University Hospital, Denmark, a tertiary referral center for EPTB in the Region of Southern Denmark. Patients were included between April 2016 and November 2018.

Eligible participants were adults (≥18 years) diagnosed with EPTB, who underwent a baseline [18F]FDG-PET/CT scan and initiated antituberculous treatment. Participants who provided informed consent underwent a follow-up [18F]FDG-PET/CT scan at the end of the intensive treatment phase, defined as 8 weeks of four-drug therapy before transition to continuation therapy.

Exclusion criteria included concurrent other infections or active malignancies, uncontrolled diabetes mellitus, prolonged systemic corticosteroid therapy, or inability to cooperate for [18F]FDG-PET/CT acquisition (e.g., inability to remain still).

### Data collection

Clinical data were retrieved from the electronic patient journal and recorded in case report forms (CRFs). The CRFs were securely stored at the investigating department and electronic datasets were maintained in the database of the Department of Nuclear Medicine at Odense University Hospital.

The dataset included demographic variables (age, sex, and ethnicity), comorbidities, and disease localization. Diagnostic work-up was recorded per localization, including specimen type and microbiological results (microscopy, culture and PCR). The date of diagnosis and whether EPTB was microbiologically confirmed or clinically diagnosed were documented.

Treatment regimens were recorded including drug combinations, treatment phase durations, and total treatment length. [18F]FDG-PET/CT results were available to the treating physicians, and treatment duration was individualized at clinician discretion.

Laboratory parameters (hemoglobin, leukocytes, albumin, alanine aminotransferase, CRP) were collected at baseline and up to three follow-up timepoints (week 8, week 12, week 32). Outcomes were categorized as treatment completion, unfavorable outcome, or loss to follow-up, and recurrence was noted where applicable.

Data quality was ensured through double-entry validation and plausibility checks. Data analysis was performed using validated systems. Stored data were secured within institutional networks accessible only to authorized personnel.

### [18F]FDG-PET/CT

[18F]FDG-PET/CT scans were performed at baseline (prior to or shortly after treatment initiation and at the end of the intensive phase. Scan protocols were based on guidelines from the European Association of Nuclear Medicine (16) and followed the department's standard protocol as previously described (17). All scans included in the longitudinal maximum standard uptake values (SUVmax) analyses were acquired using GE Discovery 690 or 710 PET/CT systems (GE Healthcare, Milwaukee, WI).

CT imaging was performed as low-dose CT without intravenous contrast enhancement. CT data were reconstructed using a standard reconstruction kernel into transaxial slices with a 50 cm field of view, a matrix size of 512 × 512, a pixel size of 0.98 mm and a slice thickness of 3.75 mm. The CT acquisition was followed immediately by PET imaging using a standard skull-base- to-mid-thigh acquisition protocol comprising six or seven bed positions, depending on patient size, with an acquisition time of 2.5 min per bed position.

The PET field of view was 70 cm, and attenuation correction was based on the CT data. PET data were reconstructed into transaxial slices with a matrix size of 128 × 128 or 256 × 256 and a slice thickness of 3.75 mm using three-dimensional iterative ordered-subset expectation maximization, with varying numbers of iterations and subsets. Images were displayed in coronal, transverse and sagittal planes. Corrections for attenuation, random coincidences, dead time and normalisation were incorporated into the iterative reconstruction.

PET and fused PET/CT images were analysed using a GE Advantage Workstation version 4.4 or a GE Advantage Server 2.0 (GE Healthcare). The fused PET/CT images were interpreted by a nuclear medicine specialist.

Patients fasted for at least 6 h before [18F]FDG administration, and plasma glucose was required to be <11 mmol/L. A weight-adjusted dose of 4 MBq/kg [18F]FDG was administered, with a minimum dose of 200 MBq and a maximum dose of 400 MBq. After 30 min rest, patients were encouraged to drink 0.5–1.0 L water during the following 30 min. After voiding, PET/CT acquisition commenced 60 ± 5 min post-injection.

SUVmax were calculated *post hoc* using Advantage Server 2.0 (GE Healthcare, Milwaukee, WI) by placing a region of interest around all areas of increased [18F]FDG uptake suggestive of infection.

All [18F]FDG-PET/CT images were evaluated by experienced nuclear medicine physicians blinded to clinical data. Lesions were assessed both visually and semi-quantitatively using SUVs including SUVmax and number of metabolically active lesions. Changes between baseline and follow-up scans were recorded.

### Statistics

Descriptive statistics were used to characterize the study population. Categorical variables were reported as frequencies and percentages, while continuous variables were presented as medians with ranges (minimum–maximum). Laboratory data collected at up to four timepoints (baseline, week 8, week 12, and week 32) were analyzed using longitudinal summary statistics. Exploratory testing for trend over time comprised Cuzick's nonparametric rank test. Changes in inflammatory markers (e.g., CRP, alanine aminotransferase, leukocytes, albumin, hemoglobin) over time were visualized using line plots. CRP dynamics, in particular, were explored across these timepoints to identify treatment response trends. We performed a complete-case analysis and did not impute missing data. Hypothesis testing was done for exploratory and hypothesis-generating purposes only. To account conservatively for multiple testing, the significance level was set to 0.001. Analyses were performed using StataNow/BE 19.5 (StataCorp, College Station, TX, USA).

## Results

### Study population

A total of 17 patients with EPTB were included in the study (Table 1). The median age was 40.6 years (range: 30–85.3) among females and 33.6 years (range: 30.6–47.0) among males, and 58.8% (*n* = 10) were female. The majority of participants were of Asian (52.9%) and African (35.3%) descent. Most patients (76.5%) were diagnosed in 2018. Comorbidities were present in 41.2% (*n* = 7), including cardiovascular disease, well-controlled diabetes, and a history of previously treated malignancies.

**Table 1:** Demographics and clinical characteristics.

Variable	Subgroup/Timepoint	Descriptive statistics (*N* = 17)
Sex	Female	10 (58.8%)
Age	Female	40.6 (30–85.3)
	Male	33.6 (30.6–47)
Ethnicity	Asian	9 (52.9%)
	African	6 (35.3%)
	European	2 (11.8%)
Year of diagnosis	2016	1 (5.9%)
	2017	3 (17.7%)
	2018	13 (76.5%)
Comorbidity	Yes	7 (41.2%)

The most common clinical presentation was lymphadenitis (*n* = 10), followed by gastrointestinal involvement (*n* = 2), spondylodiscitis (*n* = 2), psoas abscess (*n* = 1), cutaneous TB (*n* = 1), and ocular TB (*n* = 1). Relevant material was obtained from all patients. *M. tuberculosis* was confirmed by positive culture in eight patients (47%) and by PCR in an additional four patients.

### [18F]FDG-PET/CT findings

Baseline and follow-up [18F]FDG-PET/CT scans were available for all patients. Lesion sites included lymph nodes, bones, the pleura, the gastrointestinal tract, and the CNS. [18F]FDG-PET/CT scans identified metabolically active disease in all participants at baseline, with a marked reduction in [18F]FDG uptake observed at follow-up in most cases (Figure 1, Table 2). The number of hypermetabolic lesions and SUVmax generally declined after the intensive treatment phase. However, in three out of 14 patients (21.4%), namely patients 5, 9, and 14, the uptake did not decline sufficiently in all lesions.

**Figure 1:**
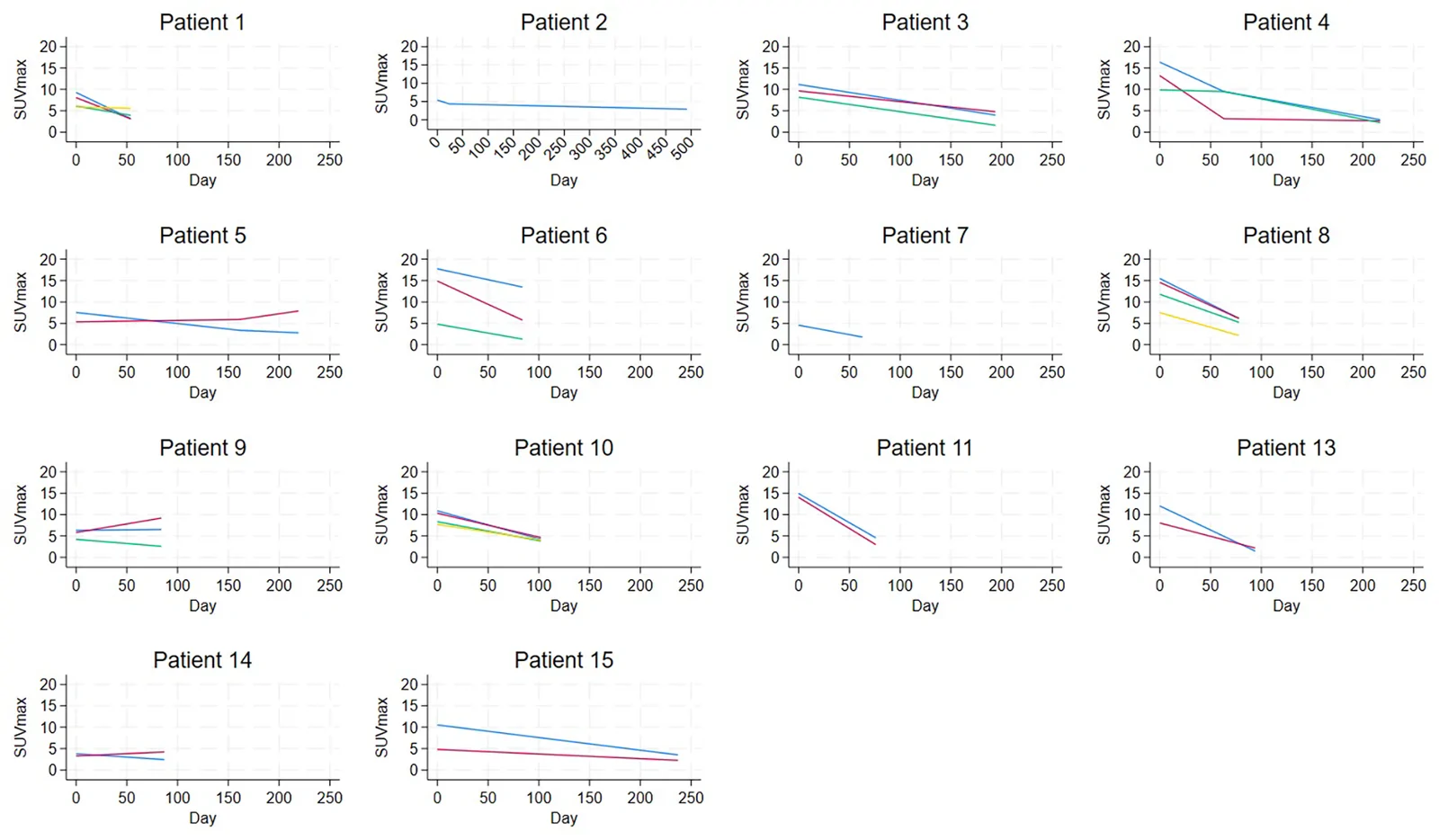
Line plots of SUVmax over time by patient. Red: lesion 1. Blue: lesion 2. Green: lesion 3. Yellow: lesion 4.

**Table 2:** Individual patient data listing for SUVmax. Increases in SUVmax compared to previous scanning are highlighted in *italic*. Patients number 12, 16, and 17 did not provide SUVmax data because they were scanned at another hospital with a different scan-type in terms of in scanner acquisition protocols and institutional standards.

Patient ID	Lesion no.	Start of treatment	Week 8	Switch to maintenance therapy	End of treatment
1	1	9.28	3.13	ND	ND
	2	8.06	3.1	ND	ND
	3	6.1	3.93	ND	ND
	4	5.95	5.55	ND	ND
2	1	5.38	4.36	2.91	ND
3	1	11.15	3.98	ND	ND
	2	9.64	4.77	ND	ND
	3	8.17	1.6	ND	ND
4	1	16.37	9.51	2.91	ND
	2	13.22	3.15	2.62	ND
	3	9.88	9.49	2.22	ND
5	1	7.55	3.38	2.8	ND
	2	5.36	*5*.*9*	*7.91*	ND
6	1	17.76	13.49	ND	ND
	2	14.91	5.75	ND	ND
	3	4.82	1.32	ND	ND
7	1	4.58	1.8	ND	ND
8	1	15.5	6.16	ND	ND
	2	14.58	6.16	ND	ND
	3	11.81	5.23	ND	ND
	4	7.53	2.15	ND	ND
9	1	6.32	*6*.*52*	ND	ND
	2	5.81	*9*.*19*	ND	ND
	3	4.22	2.58	ND	ND
10	1	10.89	4.2	ND	ND
	2	10.32	4.64	ND	ND
	3	8.35	3.83	ND	ND
	4	7.7	4.08	ND	ND
11	1	14.95	4.59	ND	ND
	2	14.04	2.98	ND	ND
12	ND	ND	ND	ND	ND
13	1	12.02	1.46	ND	ND
	2	8.04	2.18	ND	ND
14	1	3.8	2.43	ND	ND
	2	3.27	4.22	ND	ND
15	1	10.53	3.53	ND	ND
	2	4.81	2.26	ND	ND
16	ND	ND	ND	ND	ND
17	ND	ND	ND	ND	ND

ND, not determined.

### Laboratory trends

Hemoglobin and albumin levels remained broadly stable throughout follow-up (Table 3). Leukocyte counts showed a non-significant downward trend, while median alanine aminotransferase levels varied between 17 U/L and 23 U/L over time. Median CRP levels (reference: <5 mg/L) declined during treatment (*p* = 0.075), although values fluctuated across follow-up and the number of available measurements varied between visits. Figure 2 shows Patient 1 who experienced a decrease from 190 to 3.4 mg/L, Figure 3 for all remaining patients. CRP reduction was observed in 13 out of 17 patients (76.5%), CRP increase in patients 11, 13, 14, and 17. We observed a reduction of both CRP and SUVmax in 9 out of 14 patients (64.3%) and an increase in both in one patient (patient 14). There were two patients with an increase in CRP and a decrease in SUVmax (patients 11 and 13) and vice versa (patients 5 and 9).

**Table 3:** Longitudinal laboratory parameters of patients with extrapulmonary tuberculosis. Values are median (range).

Parameter	Baseline (*n* = 17)	Week 8 (*n* = 17)	Week 12 (*n* = 8)	Week 32 (*n* = 13)	*P*-value
Hemoglobin (mmol/L)	8 (5.8–10.7)	7.9 (6.9–9.7)	7.8 (6.8–10.1)	7.7 (6.4–9.7)	0.92
Leukocytes (×10⁹/L)	7.2 (4.0–9.8)	5.6 (2.1–8.9)	6.4 (4.1–10.6)	5.5 (2.6–9.6)	0.28
Albumin (g/L)	43 (27–49)	43 (34–49)	44.5 (41–50)	45 (34–52)	0.23
Alanine aminotransferase (U/L)	17 (6–83)	23 (13–115)	21.5 (18–109)	19 (15–40)	0.64
C-reactive protein (mg/L)	12 (0.3–190)	3.6 (0.9–20)	7.3 (0.9–13)	3.1 (0.8–74)	0.075

**Figure 2:**
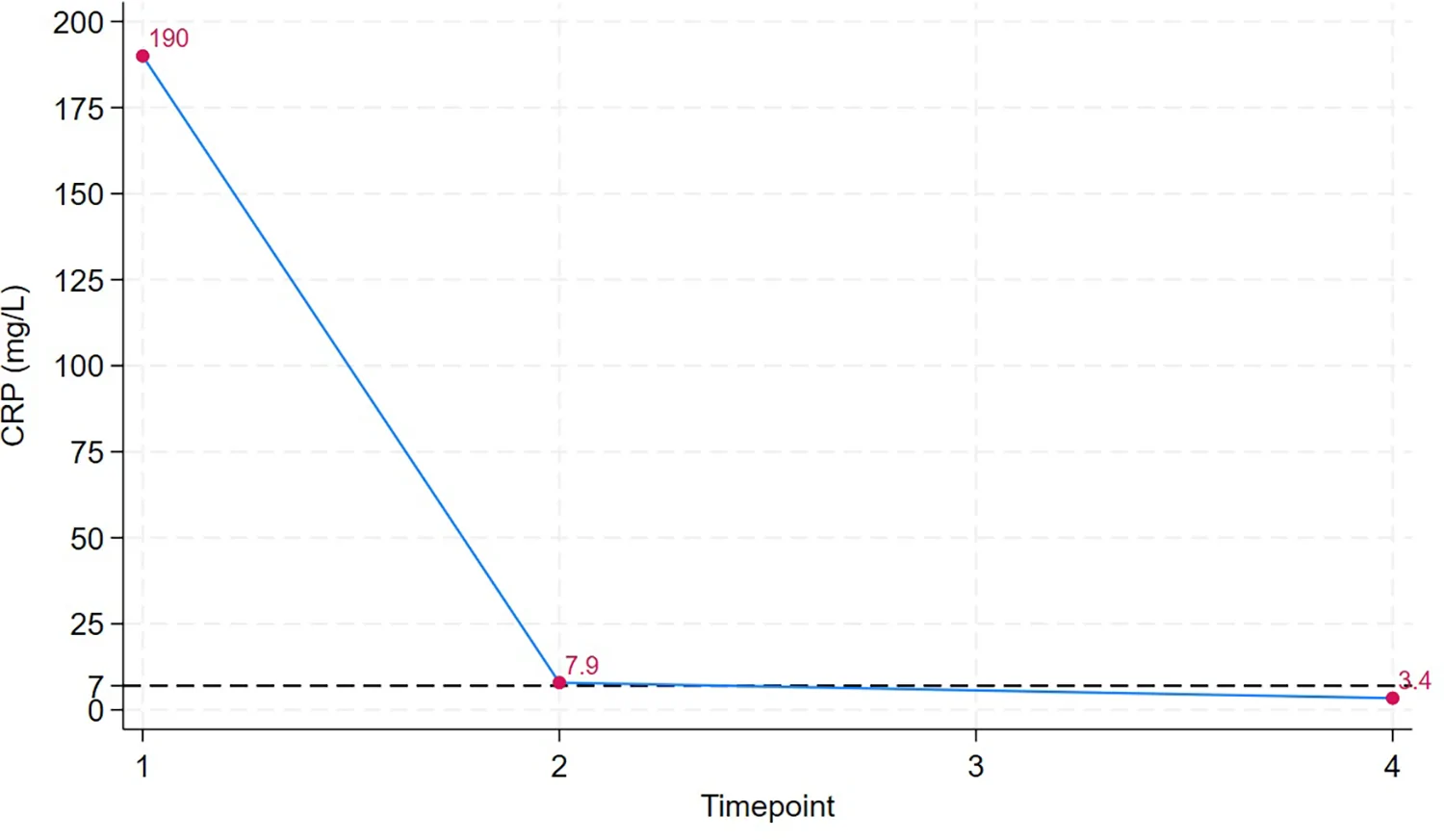
Observed CRP values (mg/L) for patient 1. Timepoints 1–4 represent start of treatment, week 8, switch to maintenance therapy, and end of treatment, respectively. The dashed line indicates a benchmark of 7 mg/L.

**Figure 3:**
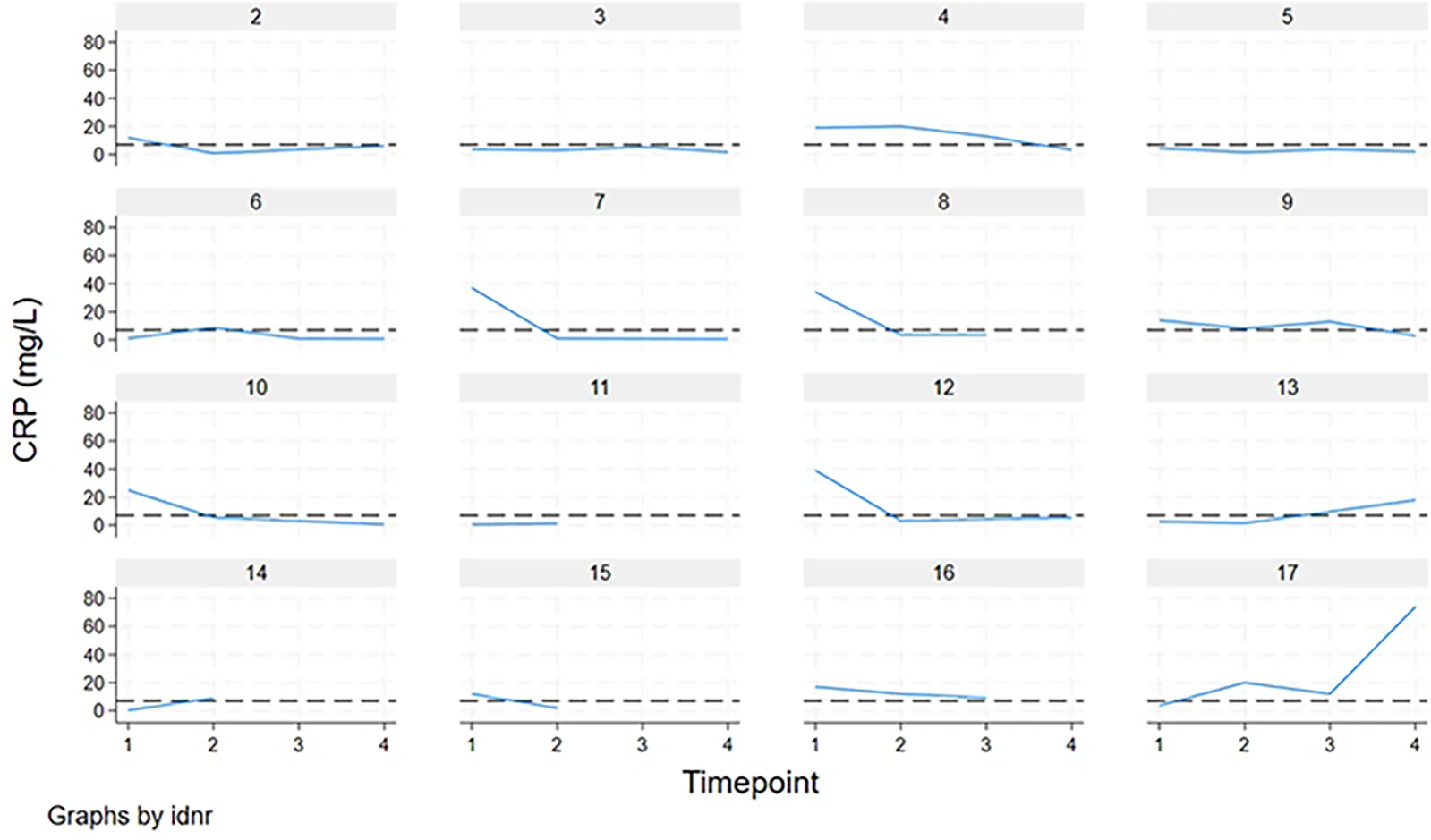
Observed CRP values (mg/L) for the remaining 16 patients. Timepoints 1–4 represent start of treatment, week 8, switch to maintenance therapy, and end of treatment, respectively. The dashed line indicates a benchmark of 7 mg/L.

### Subgroup analysis

CRP kinetics categorized patients into four groups based on thresholds of 7 mg/L at baseline and week 8. Six out of 17 patients (35.3%) started with CRP ≥ 7 mg/L and decreased below this threshold by week 8. Four patients (23.5%) had persistent elevation ≥ 7 mg/L, while another four patients (23.5%) had low CRP levels throughout. Three patients (17.6%) started with CRP < 7 mg/L and elevated beyond this benchmark in week 8.

Patients with initially elevated CRP and persistent metabolic activity on [18F]FDG-PET/CT were flagged as possible candidates for extended treatment monitoring.

### Treatment and outcome

All patients were initiated on standard four-drug antituberculous therapy (isoniazid, rifampicin, pyrazinamide, and ethambutol). In three patients, moxifloxacin was added either due to suspected isoniazid resistance following prior treatment for latent TB infection or due to suboptimal clinical response in the absence of drug susceptibility test results.

The continuation phase was extended in seven patients (patients 10–16), with a total treatment duration ranging from 37.3 to 44.6 weeks. In five patients, prolongation was primarily based on persistent symptoms and overall clinical assessment. In Patients 11 and 14, persistent [18F]FDG uptake at the end of the intensive phase (week 8) also contributed to treatment prolongation, resulting in total treatment durations of 38.0 and 40.3 weeks, respectively. Conversely, insufficiently declining uptake in Patients 5 and 9 did not lead to treatment prolongation. Treatment decisions were individualised and were not based on predefined PET/CT criteria.

All patients completed the full course of treatment, except for one patient who transferred back from Denmark to his/her home country before completion. The median treatment duration was 31.1 weeks (range: 25.9–44.6 weeks).

## Discussion

### Principal findings

In this study, we examined whether early [18F]FDG-PET/CT dynamics combined with routine laboratory markers could assist treatment monitoring in EPTB. Two main findings emerged. First, metabolically active disease was present in all participants at baseline, and most showed reduction in lesion number and SUVmax after the intensive treatment phase; nonetheless, a subset showed persistent metabolic activity. Second, CRP decreased markedly during early therapy and broadly paralleled [18F]FDG-PET/CT findings while a minority displayed discordant or persistently elevated values.

These observations suggest that integrating trajectories from imaging and biomarkers may provide early signals of therapeutic response and identify patients who may benefit from closer follow-up.

However, treatment prolongation was not consistently aligned with [18F]FDG-PET/CT findings, reflecting uncertainty regarding the clinical interpretation of residual [18F]FDG uptake. [18F]FDG uptake is non-specific and may persist due to inflammatory activity or tissue repair, limiting its ability to distinguish active infection from post-treatment changes (18).

### Comparison with previous studies

Our results align with previous evidence showing that [18F]FDG-PET/CT can sensitively detect metabolic changes during antituberculous therapy in EPTB, but that residual uptake often persists despite completion of treatment (12–15, 19, 20). In a prospective observational cohort of *n* = 147 HIV-negative adults with EPTB, serial [18F]FDG-PET/CT scans demonstrated a progressive reduction in lesion number and SUVmax over time, but the majority of patients (≈64%) remained PET-positive at the end of standard, recommended therapy, limiting its current utility as a definitive biomarker of cure or a decision tool for treatment duration (19). In HIV-infected patients with EPTB, metabolic response by 2 months was heterogeneous, and only about half of patients achieved a complete metabolic response at therapy completion, perhaps attributable to sustained metabolic activity fueled by the coinfection with HIV (21). Several smaller prospective studies, including a cohort of 25 predominantly lymph node and bone EPTB cases, report significant declines in SUVmax during therapy but substantial overlap between responders and non-responders (22). Retrospective assessments that included both pulmonary and extrapulmonary cases have noted discordances between anatomical and metabolic responses on follow-up [18F]FDG-PET/CT, with some lesions remaining metabolically active despite clinical improvement (23). Although most studies focus on changes in SUVmax, there is a growing recognition that more comprehensive quantitative metrics may better capture global disease burden and correlate with outcomes, as suggested by early analyses of whole lung PET metrics in TB cohorts (24). Collectively, these studies support the sensitivity of [18F]FDG-PET/CT for treatment monitoring in EPTB but underscore that standardized response criteria and larger cohorts are required to validate [18F]FDG-PET/CT thresholds that meaningfully predict treatment success or inform alterations in therapy.

Randomized data from pulmonary TB further indicated that PET/CT-guided stratification could identify patients at higher relapse risk. The authors concluded that residual metabolic activity and lesion volume may predict relapse risk and identify patients less suitable for treatment shortening (4-month vs. 6-month) (25). Although these findings are promising, they are derived from pulmonary disease and may not be directly generalizable to EPTB.

### Interpretation and clinical implications

Overall, our study supports [18F]FDG-PET/CT as potential adjunct tool for early response assessment in EPTB, particularly in patients with unclear clinical response. Future research should prioritize prospective study designs with predefined [18F]FDG-PET/CT response metrics and consistent scan timing. The standardized uptake value (SUV) is the primary quantitative parameter in PET, reflecting [18F]FDG uptake within a defined region or volume of interest normalized to injected dose and patient body metrics. It is simple to obtain, requires no blood sampling, and correlates with glucose metabolism. Commonly used measures include SUVmax and SUVmean (26).

However, SUV is influenced by biological and technical factors. Body composition affects accuracy, as normalization to body weight may overestimate metabolic activity in obese patients. In addition, SUVmax may overestimate uptake in heterogeneous lesions, whereas SUVmean is more representative but less reproducible and more operator-dependent. Despite these limitations, SUV remains widely used for treatment response assessment in both oncological and non-oncological diseases (27).

To overcome the limitations of lesion-based metrics, composite measures integrating metabolic activity and volume, such as total lesion glycolysis, have been introduced. Semi-automated segmentation enables calculation of metabolically active volume and corrected SUVmean, which are combined into a global disease burden metric by summation across all lesions. This approach offers a reproducible and efficient method for quantifying systemic disease and assessing treatment response, but the methods are more laborious and time-consuming and still much less validated in clinical contexts.

### Strengths and limitations

The main limitation is the small sample size, resulting in limited precision and wide uncertainty around subgroup patterns. Selection bias is possible, as patients referred for [18F]FDG-PET/CT may represent more complex or disseminated EPTB cases. A further limitation is the lack of standardisation across PET/CT scanners and protocols, which excluded three patients from the longitudinal SUVmax analysis and may limit reproducibility across sites. Although [18F]FDG-PET/CT interpretation was blinded to clinical data, [18F]FDG uptake is non-specific and may overestimate ongoing disease activity. As [18F]FDG-PET/CT results were available to the treating physicians, incorporation bias cannot be excluded. Imaging findings contributed to treatment prolongation in two patients but did not consistently correspond with treatment duration across the cohort. Consequently, this study cannot determine whether PET/CT-guided treatment decisions improve outcomes, and the findings should primarily be interpreted as describing metabolic changes during therapy. Finally, the data were collected in 2016–2018, and although [18F]FDG-PET/CT is much more widely available today and technology has evolved since then with faster and more sensitive, digital scanners imaging criteria for treatment-response assessment in EPTB remain lacking.

## Conclusion

Serial [18F]FDG-PET/CT demonstrated changes in metabolic activity during EPTB treatment, with reductions in lesion number and SUVmax in most patients. Declining SUVmax and lesion counts generally corresponded to decreasing CRP, suggesting that combined imaging and inflammatory marker trajectories may assist early treatment monitoring. Nevertheless, residual [18F]FDG uptake remained common and its clinical significance is uncertain. Larger prospective studies with standardized response criteria, volumetric PET measures and long-term clinical outcomes are needed to define the role of [18F]FDG-PET/CT in treatment monitoring of EPTB.

## Data Availability

The raw data supporting the conclusions of this article will be made available by the authors, without undue reservation.
